# On the Signs of Pulmonary Tubercles

**Published:** 1829-04-01

**Authors:** 


### ON THE SIGNS OF PULMONARY TUBERCLES.

One of the best, if not the very best papers in the first Number of the "MIDLAND REPORTER," bears the above title —and is professed to be by one of the Editors. It bears intrinsic marks of its parent, who has turned out to be Dr. Hastings. After some appropriate introductory remarks, the author lays before us the old method of diagnosis, as applied to tubercular consumption, by the heads of our profession, in order to prove that the said methods were inadequate, and inferior to those which modern investigation has brought to light.

"OLD METHOD OF DIAGNOSIS."

"Tuberculous Consumption has always been considered a very insidious disease, and often makes considerable progress before any important affection is supposed to exist. The cough, at the commencement, is hardly so considerable as to claim notice; while the extenuated habit and loss of strength, with which it is attended, are often ascribed to other causes. But there is always some presumption that these are the incipient state of the Tuberculous Phthisis, when they take place between the age of fifteen and twenty-five, and are found to continue for some time, notwithstanding the use of those means which are in general successful in Catarrh. But the symptoms mentioned above, always give strong suspicion of Tuberculous Phthisis, when they occur with those who are of that fair complexion and delicate make, which is very common with such as are of a scrofulous constitution.

"There is also reason for suspecting Tuberculous Phthisis, where the symptoms above-mentioned have begun without any obvious exciting cause; such as injuries to the breast; where the pain, when it does take place, is not fixed to any particular spot; when dyspnœa does not occur, or, if occurring, is only distressing upon motion of, or any considerable exertion on the part of, the patient.

"But above all marks, the Tuberculous Phthisis is characterized by the peculiarity of the cough, which takes place both at its commencement and during its course. The cough which attends the Tuberculous, is, as has already been said, of the short tickling kind, denominated a Tussicula. For a long time, it seems to proceed from some accidental irritation, and is attended either with no expectoration, or merely with the expectoration of a small quantity of mucus. Where a more copious expectoration does happen, it is neither viscid mucus, nor purulent matter mixed with mucus. The matter expectorated is often clear and limpid, with a reddish tint; and is rather a bloody sanies than even ill-conditioned pus."

Such are the rules laid down by the late Dr. Duncan, who has been far more precise and correct than any of our countrymen on this point. Yet every physician must be well aware how very frequently the whole of the foregoing signs are absent, with the exception of emaciation. The first of the following cases, which we shall much abridge, will be found interesting in several points of view, besides affording an illustration of the remark just made.

*Case* 1. W. M., aged 24 years, of pale complexion, was received into the WORCESTER INFIRMARY, on the 22d May, 1819. Complained of faintness, difficulty of breathing on making any exertion, giddiness, but very little cough. He had no expectoration—pulse 106, and weak —tongue clean—bowels costive—considerable emaciation. Had been ill 13 weeks. The disease commenced with sickness and vomiting, which continued more or less up to the time of admission. Blister to the epigastrium—saline aperients. During the next 6 days he rather improved; but the sickness and vomiting continued. This last affection remained obstinate till his death—and he never exhibited any symptom of hectic fever till the last.

"*Dissection.*—The Membrane lining the Epiglottis, Larynx, and Trachea, was quite healthy. There was no fluid effused in the Bronchial Cells, and the Mucous Membrane lining them, had a perfectly natural appearance. The pleura, covering the superior Lobe of each Lung, was adhering to the Costal Pleura, but in every other part of the Thorax, this Membrane was healthy; the adhesions were evidently not of recent formation. The Parenchymatous structure of the Lungs was much diseased, particularly the superior Lobes of this organ. On cutting into this portion of the Lungs, we found it, in short, to consist of one entire mass of hardened Tuberculous matter; and all appearance of the usual Cellular tissue of the Lungs was, in this part, destroyed; there was not any part of this large Tuberculous Mass in a softened state, indeed, it was every where harder than Tuberculous Matter is generally found. The inferior Lobes of the Lungs were also thickly studded with Tubercles, none of which had softened, but they had not, in these parts, coalesced to the same degree as in the Upper Lobes, for we could, in these parts, recognise the Tubercles as imbedded in the Cellular Tissue of the organ, which, as was before remarked, was not the case in the Upper Lobes. It is also worthy of observation, that the Cellular Membrane had quite a healthy appearance; it had its usual spongy feel, and was not at all inflamed or thickened; neither was any fluid, either serous or sanguinolent, effused into it: it was indeed, of a pale colour.

"The Heart was small and its fibres pale, but there was no other deviation from its usual appearance.

"*Abdomen.*—The Stomach was carefully examined, but we could not discover any tendency to Ulceration of the mucous surface, nor any other diseased state of this organ. The small Intestines appeared quite healthy. The Mucous Membrane of the large Intestines, was, in some places, slightly thickened, but there was no increased redness of any part of this tissue. The Mesenteric Glands were healthy. The Peritoneum was not at all diseased. The Liver was larger than natural, and of a pale colour, apparently from the deposition in its texture of Tuberculous Matter. There was nothing remarkable in the other Abdominal Viscera, nor in those of the Pelvis."

In this remarkable case, the disease (phthisis) may be said to have passed through its course without exhibiting any of the usual symptoms of that malady. The gastric affection appeared, and naturally enough, to the author, to be the cause of the sickness and vomiting. The slight cough was attributed to gastric irritation, and both it and the dyspnœa vanished after a few days residence in the hospital. Yet dissection shewed the whole of the disease to be in the lungs, and the stomach and bowels to be healthy.

"In seeking an explanation of this singular fact, the entire absence of Pulmonic symptoms, when so much disease was existing in the ogan itself, we must keep in view two important circumstances. First,—that although the Tuberculous deposition had proceeded to so great an extent, the usual process of softening had not taken place; the Tubercles were every where hard. And secondly,—that there was not, in any part of the tissue of the Lungs, any vestige of inflammatory action; the portions of Lung that were free from Tubercles, were quite healthy.

"Now it must, I think, be admitted, that all the active Pulmonary affections in Phthisis Pulmonalis, either arise from the softening of Tubercles, or from inflammation of the tissues of the Lung; but as neither of these existed in M.'s case, the usual symptoms were not produced. Hence arose the difficulty of the Diagnosis, for M. did not die in the manner usual in Pulmonary Consumption, by the softening of Tubercles; but these foreign bodies, by gradual accretion, filled up the structure of the Lungs, and incapacitated them for converting a sufficient quantity of the chylous parts of the food into blood, to nourish the system. The irritability of the Stomach, we may designate, as one of those instances of sympathy which are so frequently occurring in the human system; and the consent, in this instance, is of a very intelligible nature; for, as the Lungs could no longer assimilate any new fluids that might be sent to them, the Stomach ceased to digest, from want of its due supply of blood."

The author thinks that this case is fatal to the doctrine of Broussais, who makes tubercles to depend on inflammation. The best pathologists in this country, as well as on the Continent, consider the growth of tubercles as independent of inflammation, but as greatly accelerated by the supervention of that state. The case above-detailed is not conclusive. Inflammation might have given origin to the tubercles (supposing it capable of doing so) and yet the inflammation might be cured, and the traces of it in the lungs obliterated, while the tubercles remained. It is remarked by the author, that had he been then acquainted with auscultation and percussion, he would not have fallen into the false diagnosis above-mentioned. The treatment might not indeed have been more successful; but it would have been more rational.

*Case* 2.—Chronic Peritonitis—Pulmonary Excavation. Dr. ——, was consulted in the case of a young gentleman aged 14 years, who had, for several weeks been affected with pain and tenderness of the abdomen, particularly low down, the bowels being irregular—pulse quick and hard—tongue morbidly red—progressive emaciation — high coloured urine—abdomen fuller than natural, as well as tender, but without fluctuation—ankles œdematous in the evening. No pectoral symptoms had been complained of. He breathed free, and ascended stairs without inconvenience. The abdominal symptoms, in this case, engrossed the reporter's attention, and he attributed them to disease of the mesentery combined with chronic peritonitis. Leeches were applied every third day—a large blister was next put over the abdomen, and the diet was regulated. By these means the symptoms were mitigated—the pain, tenderness, and tension being much relieved; but the young gentleman did not improve in other respects—the weakness and emaciation progressively increasing. Evening fever came on, with morning perspirations, and he died in six weeks. He never coughed, nor exhibited any symptoms that would lead to suspicion of pulmonary disease.

*Dissection.* "Abdomen.—On opening this cavity, the Intestines were found generally adhering together, and to the Peritoneal Lining of the Abdominal Muscles. There was not discovered any serous effusion, and the adhesions did not appear to be of very recent origin. In several parts of the Peritonæum, small Tuberculous Depositions were seen, more particularly on the surface of the Serous Membrane, which covers the Liver. The Liver was paler than natural, and enlarged. Some of the Mesenteric Glands were enlarged, but none of them to any remarkable degree. The Stomach was healthy; nothing worthy of notice appeared in the Mucous Membrane. The Mucous Membrane of the small and large intestines was not diseased, excepting that lining the Cæcum, which was in some degree thickened.

"*Thorax.*—The Mucous Membrane lining the Larynx, Trachea, and Bronchia, was free from disease. There was softened Tuberculous Matter in the left Bronchus. The right cavity of the Thorax contained a small quantity of serous fluid, but there were no adhesions of the Pleura. The Cellular Membrane of the right Lung shewed no mark of inflammatory action. It had lost none of its usual elasticity, nor was there any congestion of its Blood Vessels. There were some Tubercles, but none of them of a large size, cheesy in their nature; this was more especially the case in the superior Lobe of the Lung. The left cavity of the Thorax contained some serous fluid, and there was very general adhesion of the Pleura, but not of recent occurrence. The superior Lobe of this Lung was almost entirely occupied by Tubercles, which had coalesced, and the centre of them had softened, forming a cavity nearly full of Tuberculous Matter, which communicated with the Bronchia. The inferior Lobe of this Lung also contained many Tubercles, some of large size, but none of these had softened."

The above case points out, in a very striking manner, how uncertain are the *functional* symptoms as compared with the *physical signs* of pulmonary disease. Although neither the patient nor friends observed any cough in this case, yet the reporter did occasionally notice a very trifling dry cough during the last fortnight of the young gentleman's illness. No expectoration, however, was ever detected.

If it be said—and doubtless it will be said—that a knowledge of the pulmonary disease would have made no difference in the final issue;—we reply that after granting all this, (which, by the way, is not quite proved, since we find marks of inflammation in that side of the chest which was most disorganized) there yet remains an important consideration to be taken into account. Is it likely that the parents and friends of this young gentleman never once asked the Doctor what was the nature of the patient's disease? What did he say to such enquiries? "Disease in the abdomen." But, on dissection, the cause of death was found to be in the chest!! If any of the friends were present at the examination, they must have formed a high opinion of the Doctor's discernment, and would, of course, employ him again on all alarming occasions. If no friends were present, the Doctor must have candidly confessed the mistake—or told a falsehood, and gone home with all the pleasant reflexions of self-degradation!

It was only yesterday, that we had an opportunity of seeing an exquisite specimen of tracheal and bronchial inflammation in a child. A practitioner, who was an attentive observer, and who studied his profession rather than scandal and abuse, stated what the disease was; but the friends called in another practitioner, who employed himself more in reforming the profession than curing the bodies of his patients. This *gentleman* pronounced at once that the other practitioner was wrong, and that the disease was in the *head*. The patient died, and the radical reformer just raised the sternum, and shut it down close again, affirming that there was no disease there! The first practitioner obtained leave to re-examine the body, and the result was, as stated above, the discovery of intense tracheitis and bronchitis!

Let us now reverse the picture, and shew that the Doctor is sometimes *agreeably* deceived in his diagnosis of pulmonary disease.

*Case* 3. SUPPOSED PULMONARY PHTHISIS, WITH SEVERE SYMPTOMS, TERMINATING FAVOURABLY. In November, the Doctor was called to a young lady who had been affected, for six months, with cough and emaciation, but without any expectoration. There was heat of face and hands in the evening, preceded by shivering—morning perspiration—pulse from 100 to 120—pain in the left side of the chest, aggravated by coughing—*tongue loaded, fauces red, stools offensive and clay-coloured*—breathing hurried on the least exertion—catamenia apparently regular. The lady was confined to a regulated temperature, in two rooms, for the space of seven or eight months, while leeches were frequently applied to the side—a seton established there—calomel and jalap given every third morning, and colchicum three times a day—vegetable diet—abstinence from animal food. These means made not the slightest impression on the complaint—on the contrary, all the symptoms were increased, and hæmoptysis was added to the list! The treatment was, however, continued; and, as the Summer advanced, the young lady began to rally, and a trip to Malvern, with horse exercise, established the cure.

With all due deference to the talented reporter, we consider the above case as one of disorder of the digestive organs and uterine system, and not one of real pulmonary disease at all. That the chylopoietic functions were in a very bad state, is evident from the symptoms themselves above-detailed—and that the catamenial functions are often very much disordered, while they are *said* to be regular, is well known to every practitioner. That the regulated temperature, bleeding, seton, and vegetable diet, exasperated the complaints there can be no doubt—and that the approach of Summer, the air of Malvern, the horse-exercise, and DAME NATURE, did more towards the cure than all the means and medicines above enumerated, will probably be surmised by most of our readers when they peruse the case.

We shall introduce the particulars of one more example of the uncertainty in question.

*Case* 4. "SEVERE SYMPTOMS OF PULMONARY PHTHISIS IN A YOUNG LADY TERMINATING FAVOURABLY." A young lady aged 14 years, tall and slender, had enjoyed good health till after an attack of measles, in April, 1820. The disease was severe, and the inflammatory fever ran high, the cough being very harrassing. The severity of the symptoms appeared to authorise strict measures. Blood was twice taken from the arm, and once locally, before the eruption went off. The pulmonary affection did not subside with the disappearance of the eruption, and repeated venesections were employed, with antimony and the usual means. At length, the prominent symptoms were subdued, and she was able to go to the sea, where she amended considerably. But the return of Winter brought a return of all the common symptoms of approaching phthisis, and slight hæmoptysis supervened. Venesection—digitalis—antimony—confinement in a regulated temperature—abstinence from all animal food. The hæmorrhage ceased; "but to it succeeded a more frequent cough, a pretty copious muco-purulent expectoration, and a decided increase of the evening paroxysm of fever." The emaciation proceeded, and there was pain in the left side of the chest. A seton was now inserted—the vegetable diet continued—and blue pill, digitalis and colchicum were given. Throughout the Winter and Spring months, all the symptoms went on from bad to worse, and the most desponding views were taken of the case.

"Happily, towards the end of May, there was a mitigation of some of the more dangerous symptoms. The breathing was less hurried on exertion; the cough was not so troublesome, and the expectoration diminished in quantity. The pulse, too, became slower, and there was some little return of flesh. As the Summer advanced, it was truly pleasing to observe, that all the worst symptoms vanished, and, by the close of the month of June, the Young Lady was almost in a convalescent state, having been enabled, early in the above month, to leave her apartments, and to take carriage-exercise in the open air. During the Summer, the flesh and strength returned; and though the cough was still occasionally troublesome, it was not accompanied by pain in the side; nor was there any evening fever. An excursion to the Sea side, in the Autumn, seemed still further to confirm this amendment, and she returned home, in the beginning of October, in tolerable health, though thin."

We confess that we entertain great doubts respecting the *good* effects of the regulated temperature, digitalis, and starvation in the above case. In fact, the young lady got worse under this treatment, and we hesitate to give credit to any medicine but the change of season. To one conclusion, however, we may safely come—that the disease was not tubercular phthisis, otherwise so favourable an issue would not have taken place. The two cases shew how uncertain are functional symptoms, unaided by physical signs. The author promises us another paper on the subject, and we have no doubt that *it* will embrace the application of auscultation and percussion as auxiliaries to the other and imperfect means of diagnosis.*

* After this had gone to press, we received the 3d Number of the MIDLAND REPORTER, in which Dr. Hastings has given the finale of his paper. We shall notice it on another occasion. In the mean time, we congratulate the zealous editor on the increasing list of his subscribers and contributors, while we wish his journal every degree of success.

